# Teaching Iramuteq for use in qualitative research according to YouTube videos: an exploratory-descriptive study

**DOI:** 10.1590/1980-220X-REEUSP-2021-0396

**Published:** 2022-02-04

**Authors:** Samira Silva Santos Soares, Carolina Cabral Pereira da Costa, Eloá Carneiro Carvalho, Ana Beatriz Azevedo Queiroz, Patrícia Lima Pereira Peres, Norma Valéria Dantas de Oliveira Souza

**Affiliations:** 1 Universidade do Estado do Rio de Janeiro, Faculdade de Enfermagem, Programa de Pós-Graduação em Enfermagem, Rio de Janeiro, RJ, Brazil.; 2 Universidade do Estado do Rio de Janeiro, Faculdade de Enfermagem, Departamento de Enfermagem Médico-Cirúrgica, Rio de Janeiro, RJ, Brazil.; 3 Universidade do Estado do Rio de Janeiro, Faculdade de Enfermagem, Departamento de Enfermagem em Saúde Pública, Rio de Janeiro, RJ, Brazil.; 4 Universidade Federal do Rio de Janeiro, Escola de Enfermagem Anna Nery, Departamento de Enfermagem em Saúde Materno-Infantil, Programa de Pós-Graduação em Enfermagem, Rio de Janeiro, RJ, Brazil.; 5 Universidade do Estado do Rio de Janeiro, Faculdade de Enfermagem, Departamento de Enfermagem Materno-Infantil. Rio de Janeiro, RJ, Brazil.

**Keywords:** Social Media, Information, COVID-19, Qualitative Research, Teaching, Software, Medios de Comunicación Sociales, Información, COVID-19, Investigación Cualitativa, Enseñando, Programas Informático, Mídias Sociais, Informação, COVID-19, Pesquisa Qualitativa, Ensino, Software

## Abstract

**Objectives:**

to identify the contents and characteristics of videos posted on YouTube that deal with Iramuteq software use as a tool to assist qualitative research and discuss the contribution of this social network to dissemination of knowledge, especially in the context of the COVID-19 pandemic.

**Method:**

this is an exploratory-descriptive study with a qualitative approach, carried out with YouTube videos, collected in June 2021 and subjected to thematic analysis.

**Results:**

a total of 43 videos on the researched topic were identified, with the majority (69.76%) published from 2020–2021. As for content, five categories were identified.

**Conclusion:**

the videos explain about Iramuteq installation, the textual corpus preparation, the errors identified by users and methods to correct them, the lexical analysis developed by the program and how to use it. The content is presented in a theoretical and practical way through the software interface presentation. Thus, YouTube’s contribution to developing teaching and disseminating knowledge is observed, both because it constitutes itself as a source for research and because it fosters dialogues between researchers, especially in a pandemic context.

## RESUMO

**Objetivos:**

identificar os conteúdos e características dos vídeos postados na rede social YouTube que tratam do uso do *software* Iramuteq *c*omo ferramenta para auxiliar as pesquisas qualitativas e discutir a contribuição desta rede social para a difusão do conhecimento, especialmente no contexto da pandemia de COVID-19.

**Método:**

estudo exploratório-descritivo de abordagem qualitativa, realizado com vídeos do YouTube, coletados em junho de 2021 e submetidos à análise temática.

**Resultados:**

foram identificados 43 vídeos sobre a temática pesquisada, sendo a maioria (69,76%) publicado no período de 2020–2021. Quanto ao conteúdo, identificaram-se cinco categorias.

**Conclusão:**

os vídeos explanam sobre a instalação do Iramuteq, o preparo do *corpus* textual, os erros identificados por usuários do *software* e métodos para corrigi-los, as análises lexicais desenvolvidas pelo programa e como utilizá-lo. O conteúdo é apresentado de forma teórica e prática por meio da apresentação da *interface* do *software*. Assim, observa-se a contribuição do YouTube para o desenvolvimento do ensino e difusão do conhecimento, tanto por constituir-se como uma fonte para pesquisa como também por suscitar diálogos entre pesquisadores, especialmente em um contexto de pandemia.

## RESUMEN

**Objetivos:**

identificar los contenidos y características de los videos publicados en la red social YouTube que abordan el uso del software Iramuteq como herramienta para ayudar a la investigación cualitativa y discutir la contribución de esta red social a la difusión del conocimiento, especialmente en el contexto de la pandemia de COVID-19.

**Método:**

estudio exploratorio-descriptivo con abordaje cualitativo, realizado con videos de YouTube, recolectados en junio de 2021 y sometidos a análisis temático.

**Resultados:**

se identificaron 43 videos sobre el tema investigado, la mayoría (69,76%) publicados en el período 2020-2021. En cuanto al contenido, se identificaron cinco categorías.

**Conclusión:**

los videos explican sobre la instalación de Iramuteq, la preparación del corpus textual, los errores identificados por los usuarios del software y los métodos para corregirlos, el análisis léxico desarrollado por el programa y cómo utilizarlo. El contenido se presenta de forma teórica y práctica a través de la presentación de la interfaz del software. Así, se observa la contribución de YouTube al desarrollo de la docencia y la difusión del conocimiento, tanto porque se constituye como fuente de investigación como porque provoca diálogos entre investigadores, especialmente en un contexto pandémico.

## INTRODUCTION

In the field of science, there is a common dichotomy between quantitative and qualitative research, as well as the valorization of quantitative studies at the expense of scientific investigations of a qualitative nature, showing a distinguished perspective of rationality for quantitative methodological approaches. However, it is necessary to consider that, according to the phenomenon to be investigated, qualitative research can provide a relevant deepening of the reality and essence of the study’s focus, resulting in the production of critical, emancipatory knowledge committed to social transformation^(1)^. Furthermore, the alliance between qualitative and quantitative research nis an articulation that can be appropriate, favoring, in many cases, a better understanding of reality, representing gains for research and scientific production.

Considering the technological advances as well as the incorporation of tools that help the development of researches, since the 1980s, there has been a growing use of computer programs known as Computer Aided Qualitative Dada Analysis Software (CAQDAS)^(2)^, applied to qualitative studies. There are countless software available today, and the three most used in qualitative research in the health field require a license to use^(3)^. This fact can make it difficult for researchers to access these programs, especially in Brazil, where there are constant financial cuts for research.

Faced with this issue, free and open access software gain notoriety. It is the case of the *Interface de R pour les Analyses Multidimensionnelles de Texts es de Questionnaires* (Iramuteq), developed by French researcher Pierre Ratinaud in 2009 and which has been increasingly used in qualitative research in Brazil and internationally, as it has complete dictionaries in several languages^(4)^.

Iramuteq is developed in the Python language and uses functionalities provided by the statistical software R, which is also freely accessible^(3)^. In the Brazilian context, research with Iramuteq began in 2013, but a scoping review carried out from the Bank of Theses and Dissertations of the Coordination for the Improvement of Higher Education Personnel (CAPES – *Coordenação de Aperfeiçoamento de Pessoal de Nível Superior*) identified that the use of that software in qualitative research, produced within the scope of graduate programs in health, presents some inconsistencies, such as: use it as a data analysis technique; use in small volume of text; and use of the Descending Hierarchical Classification (DHC), even in the face of unsatisfactory performance, i.e., less than 75% of the material analyzed^(3)^.

Due to these inconsistencies, it is essential to know Iramuteq in depth so that the desired scientific rigor for research is achieved and that the software’s functionalities allow it to reach its complete efficiency in qualitative data management and retrieval^(2)^.

Corroborating the above, another issue that deserves to be highlighted was pointed out by a review study, in which it was identified that there is underutilization of Iramuteq’s technical resources, which limits the broader understanding of the phenomenon studied^(5)^. In this regard, it is worth considering that despite the Iramuteq interface being simple to use, the program provides different possibilities for textual and matrix analysis; in each of these, there are numerous features, parameters and results to be displayed so that these can and still need to be explored in searches. This is mainly the case for matrices, i.e., textual materials organized in spreadsheets and that make up databases to be explored, such as the results of free word recall tests^(6)^.

In this sense, it is essential to equip researchers on the software use, which can be done through on-site and/or remote training, such as through tutorial videos, synchronous and asynchronous classes on digital platforms, among other channels. It is also noteworthy that, lexical analysis using Iramuteq, in addition to streamlining data processing, especially in the face of a large textual volume, can increase methodological rigor in research, because when using its resources, researchers obtain information with statistical bases to analyze the meaning of terms/words in the corpus. This process allows for a more in-depth qualitative data analysis, as it provides transparency regarding the process of inference and analysis of the processed material, increasing the possibilities of deepening and reliability of results^(5)^.

Furthermore, it is noteworthy that the program use is not intended to remove and/or replace the researcher’s role in data analysis. On the contrary, the researcher plays a central role, taking responsible both for preparing the textual corpus to be analyzed as well as conducting data analysis based on results produced by the software^(2,3)^. In this sense, it should be noted that the textual corpus preparation is a fundamental step; after all, it refers to the moment when, after unifying all textual content to be analyzed in a single file, the researcher must carry out a critical and thorough text verification. Then, spelling errors are avoided, acronyms are standardized, synonym use is standardized, removing language vice use, among other aspects, which will make the material more robust for analysis^(6)^. However, in this process, it is emphasized that one should not interfere in the meaning of the content produced.

In addition to the above, it was empirically observed that the number of videos published online about Iramuteq has increased in recent years (2020–2021). This fact can be attributed to the context of the COVID-19 pandemic, because, instead of on-site training and courses in research groups and universities, these scenarios were replaced by virtual rooms and platforms. Furthermore, due to the fact that in this health crisis, social distancing is recommended, there was a tendency to give more visibility to social and information media, emphasizing the importance of introducing new technologies to the teaching-learning process^(7)^.

Supporting this thought, it can be considered that, when exploring the potential of the internet (global system of interconnected computer networks) and its social communication media to socialize knowledge, a revolution in the model of production, distribution and updating of knowledge and science is promoted, enshrining these devices as important strategies for disseminating information in society and in the academic sphere.

Given the context, the objective was to identify the contents and characteristics of the videos posted on YouTube that deal with Iramuteq use as a tool to assist in qualitative research and discuss the contributions of social media as knowledge dissemination strategies, especially in the context of the COVID-19 pandemic.

## METHOD

### Study Design

This is a descriptive-exploratory study with a qualitative approach, carried out on the social network YouTube (www.youtube.com), considering the COREQ tool criteria.

YouTube was chosen as it is one of the most important social media platforms for sharing videos, with free access to internet users^(8)^. In other words, the proposal of the platform chosen for data collection is in line with that adopted by the creator of Iramuteq, since, in addition to being free to access the content, it allows, through community interaction/participation, to expand its dissemination/use and improve its development based on the comments of the users mentioned in the aforementioned social network^(9)^.

### Video Selection Criteria and Data Collection

For the identification of videos and access to content, a research protocol was defined with five steps: (i) demarcation of theme, objective and guiding question; (ii) establishment of search strategy (video gathering); (iii) video analysis; (iv) data extraction; and (v) presentation and dissemination of primary results.

Data collection took place on June 8 and 9, 2021, from the combination of keywords (tags) “iramuteq” and “qualitative research”. These terms were typed in the search box available by the platform itself. Then, the following filters available on YouTube were assigned to select the videos: type of publication (video) and presentation of results (sorted by date of submission). It is noteworthy that there was no filter with a time frame, hoping to identify the largest amount of videos available.

Videos that address content related to Iramuteq and qualitative research and be in audio or subtitles in Portuguese, English or Spanish, regardless of duration and target audience (undergraduate or graduate students, in the health area or not), were included. Videos with audio problems, making it difficult to understand the content or without subtitles, which have duplicate content, those with purely commercial content, i.e., with the purpose of promoting a brand and/or selling a product, dealing with another type of research other than qualitative (e.g., bibliometrics) and had dubious content or fake news were excluded.

To verify this last criterion, the British Broadcasting Corporation recommendations were considered^(10)^. In other words, the contents of the videos were submitted to veracity verification, which adopted a scoring system of 0, 1 or 2 for each criterion (channel, content and comments), with 0 being unreliable/harmful, 1 for doubtful/questionable and 2 for reliable/looks good. In summary, after adding up the scores, the videos were classified as unreliable (0 to 2 points), doubtful (3 to 5 points) and trustworthy (6 points). This criterion, which deals with the quality of the videos, followed the same foundations adopted in similar investigations, as there is, to date, no validated and specific instrument for such an assessment^(11,12)^.

Furthermore, it is worth considering that, in order to assess the quality of information presented by the videos regarding the software use reliability, the researchers compared them with the Iramuteq tutorial guidelines, available at “Iramuteq.org”^(6)^.

**Figure 1.**
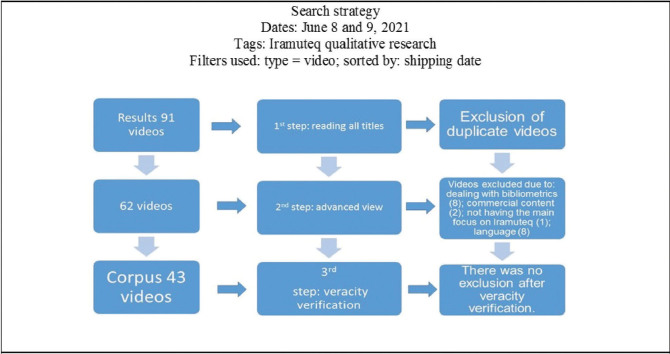
Process of constitution of the corpus. Rio de Janeiro, RJ, Brazil, 2021.

Initially, 91 videos were found from reading all titles, and with advanced visualization of the material and verification of its veracity, this resulted in 43 videos in the study corpus. This data collection and organization step was performed by two authors. The synthesis of the methodological process of constitution of the corpus is shown in Figure 1.

Next, the electronic addresses of the identified videos were selected and arranged in a Microsoft Excel 2016 spreadsheet. Thus, researchers could have unrestricted access to the material and make queries whenever necessary.

Afterwards, each video was analyzed individually, proceeding to the identification of themes, synthesis of its content and categorization. The themes and categories were initially elaborated by two researchers of the study in question. It should be noted that, in case of doubts or divergences in relation to the process of elaboration of the themes and categorization of the material, a third component carried out another verification of the process, in order to clarify doubts and enable methodological consistency in that procedure.

### Data Analysis

Data were analyzed according to indicators present in a Microsoft Excel 2016 spreadsheet. This worksheet was developed by the authors of this manuscript, in order to facilitate the tabulation of data and was adapted from previously validated instruments used in surveys with a similar profile in order to standardize information extraction^(11,12,13,14)^. Thus, the following was identified: (i) publication date and total number of views (described in the video itself); (ii) video duration time; (iii) author responsible for the publication (individual, legal or other); (iv) content approach (whether the content of Iramuteq is presented in a theoretical or practical form, if it describes all the possibilities of analysis or just one, advantages and disadvantages).

### Ethical Aspects

As it does not involve contact of any kind with the characters in the videos or the channel owners, and considering that the data used comes from an open and free website (public domain), there was no need to submit this research to the research ethics committee. However, it is noteworthy that the YouTube platform guidelines were respected^(15)^.

## RESULTS

**Figure 2.**
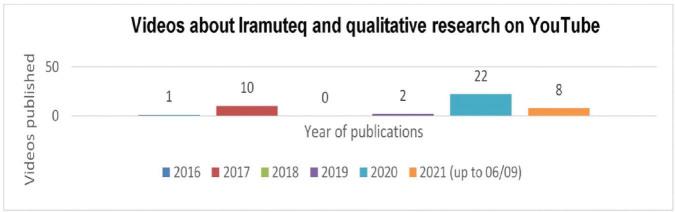
Iramuteq and qualitative research on YouTube between 2016 and 06/09/2021. Rio de Janeiro, RJ, Brazil, 2021.

The 43 selected videos were produced and published on a YouTube channel by individuals (05 professors, 02 undergraduate and 05 graduate students), academic consulting companies (08 videos) and, mainly, by groups, centers and research laboratories linked to Higher Education Institutions (23 videos). Figure 2 allows us to observe the quantity of videos published by period/year.

It is noted that the videos produced from 2020–2021 (until the date of data collection – 06/09/2021) already represented 69.76% of the material produced, significantly surpassing the quantity of videos produced in previous years.

The videos had their content characterized based on common themes and organized into five categories: *Iramuteq installation*; *Textual corpus preparation*; *Iramuteq errors*; *Iramuteq analyses*; *Iramuteq in qualitative research – short, medium and long videos*.

Short videos were considered (those with a maximum of 15 minutes), medium (material with more than 15 minutes and up to 60 minutes, i.e., the average time of one hour/class) and long duration (recordings over an hour). This subdivision was chosen only in this category, due to the volume of videos that comprise it (n = 22) and, especially, because most of them are long (n = 10), different from what occurred in all the other categories.

The number of views is a metric that establishes a relationship with the interest of YouTube users in relation to the topic presented. Table 1 shows the number of video views (up to the date of data collection) by category.

**Table 1.** Video views by category (n = 43) – Rio de Janeiro, RJ, Brazil, 2021.

Categories	Views	Total videos
Category 1: Iramuteq installation	21,353	7
Category 2: Textual corpus preparation	21,999	2
Category 3: Iramuteq errors	9,620	2
Category 4: Iramuteq analyses	56,909	10
Category 5: Iramuteq in qualitative research	74,342	22
Subcategory 5.1: Short videos	15,520	8
Subcategory 5.2: Medium videos	51,452	4
Subcategory 5.3: Long videos	7,370	10
All categories	184,223	43

### Iramuteq Installation (N = 07)

This category brings together six videos in which the authors explain the process of installing Iramuteq for the Windows^®^ operating system and only one video covers the installation on Mac^®^ (operating system developed and distributed by Apple^®^). Most videos are short (up to 15 minutes) and are intended to help people interested in using Iramuteq to properly install the program. Therefore, they rescue instructions that are presented in the Iramuteq tutorial, demonstrating in practice the step-by-step download and installation of the R software first and, later, of Iramuteq.

The videos in this category were developed by different channels and authors, namely: an academic linked to a master’s degree program in professional technological education; a professor of psychology course; a social psychology monitor linked to a research laboratory at a federal university in the south of Brazil; a research group on Transport Behavior and New Technologies; and a social psychology research laboratory at a university in the northeast of Brazil. This last video mentioned starts a series of 09 videos that are part of an introductory course to Iramuteq and have been published on YouTube since 2017.

It should also be noted that two videos in this category are published on a commercial channel that helps academics in the field of scientific methodology, providing videos about the challenges of making science. This channel is the result of an extension project of a federal university started in 2018 that became an academic consultancy in 2019 and in 2020 it started to offer services in the methodological field and courses via Telegram (cloud-based instant messaging service available for smartphones or tablets, computers and also as a web application)^(16)^.

### Textual Corpus Preparation (N = 02)

The videos in this category emphasize the textual corpus preparation so that it can be processed by Iramuteq. The corpus is the set of texts constructed by the researcher and intended to be analyzed^(6)^. Therefore, it can be formed by a set of transcribed interviews, news publications or reports, among other textual elements, chosen by the researcher according to what is intended to be investigated. It is noteworthy that it is a single text file saved in the text.txt modality, separated by command lines according to the research variables, as established by the researcher, taking into account its object of study^(6)^.

One of the videos is presented by a professor linked to a Laboratory of Studies and Practices in Psychology and Health, which proposes to articulate teaching, research and extension in psychology with an interface in health and public policies. Despite being a short video (13 minutes 23 seconds), it is didactic and presents the differentiation in the nomenclature used by Alceste and Iramuteq, it talks about the number of texts that can make up the database and how to separate these texts through lines of command or metadata. It also informs the identification number of the material (text that follows) and the characteristics (variables), which are important for the research design.

In addition to this, the video shows, through a practical example, how a corpus is prepared, whether monothematic or organized into sub-themes, and the importance of revising the text as instructed in the software tutorial in order to produce a successful analysis.

The other video in this category was produced by an academic linked to a master’s degree course in professional and technological education. Before discussing the preparation of the corpus itself, the author of the video highlights the importance of verifying that R packages are up to date, so that there are no failures/error mentions during analysis processing.

Unlike the video mentioned above and that makes up this category, in this material, the corpus is worked on in a notepad, while, in the previous one, it was presented and manipulated through a Microsoft Word^®^ document. However, at the end of the corpus preparation, it is observed that, in both videos, the documents produced are saved in UTF-8 format, which is recognized by Iramuteq. There is also clarification in the two videos about the type of textual content that can be submitted to the software and the indication of Iramuteq tutorial so that the corpus can be prepared in Libre Office^®^ or in Open Office^®^, as they are open access.

### Iramuteq Errors (N = 02)

The videos in this category emphasize methods for resolving possible Iramuteq errors. As the software uses the R statistical environment, the errors presented are usually related to the outdated R analysis packages. These, in turn, can be checked through the preferences menu, “editing” option and finally through from the “verify” button. Another aspect that is highlighted in the videos is the compatibility between the versions of these software, when producing the videos. Thus, the most recent, compatible and indicated versions were version 0.7 Alpha 2 of Iramuteq and version 3.2.3 of R.

### Iramuteq Analysis (N = 10)

The videos that make up this category, in general, talk about the various types of textual analysis that can be produced with the help of Iramuteq and only one video deals with matrix analysis. There are five videos that exclusively present each of the textual analyses, namely: textual statistics, Reinert method (which performs DHC), group specificity search/correspondence factorial analysis (CFA), similarity analysis and word cloud.

Two other videos are part of a short course by the Quality of Care and Health Education Research Group at a federal university of Rio Grande do Norte. The short course was offered in two days. Basic notions about Iramuteq were presented in the first virtual meeting and in the second meeting practical considerations/approaches in relation to the software use were presented. It is worth mentioning that each meeting lasted about 2 hours and 20 minutes.

### Iramuteq in Qualitative Research – Short, Medium and Long Videos (N = 08)

In this session, all videos deal with Iramuteq use as a tool to assist the data treatment process in qualitative research.

Among the short videos (n = 8), two were presentations of work using Iramuteq and published in scientific events that took place on the internet. One video discussed the advantages and disadvantages of using Iramuteq, and another, in addition to describing the software, also briefly presented the NVIVO and ATLAS.ti programs. The latter require a license to use; however, they offer a free trial period and are indicated to assist in qualitative research data treatment.

There are also four other videos in this subcategory, which present the first steps in Iramuteq use, i.e., they are videos with tips on how the software works and its possibilities for analysis in qualitative research.

As for medium videos (n = 4), all of them present a brief explanation of Iramuteq use and briefly discuss the types of textual analysis that the software provides. Little or no emphasis is placed on matrix analysis.

In relation to long videos (n = 10), diversity of areas and target audience for which they have been produced stand out, namely: Health (epidemiology, work, nursing), education, historical research, society and politics, engineering education, administration and accounting science. All full-length videos were published from 2020–2021.

## DISCUSSION

Since the COVID-19 pandemic was enacted and culminated in the interruption of in-person classes, numerous initiatives have been adopted, especially by public universities, their undergraduate and graduate programs (*lato* and *stricto sensu*) to promote the integration between professors and students, the updating of knowledge as well as the dissemination of science, even in the face of this new scenario^(17,18)^.

In this context, new pedagogical practices and methodologies stood out, including the production of lives, webinars, online mini-courses and various multimedia teaching materials. YouTube has become increasingly known to professors/researchers and academics, enabling them to use their knowledge to create content and make it available, helping a high number of people^(19)^. Universalization and access to technology have certainly contributed to an innovative and increasingly accessible and diversified education^(18,20)^, as can be seen in the videos that integrated this research.

Furthermore, in this pandemic moment, technology use was presented as an important tool to “bring people together and reduce distances”, thus benefiting the social collective. From a health perspective, it minimizes the risks related to SARS-CoV-2 transmission, as it replaces face-to-face meetings in relation to the educational process^(21)^, breaking the barriers of physical distance between professors and students and other people/users interested in the topics being published.

It is known that not all YouTube videos can be recommended for educational purposes due to content inconsistencies and inadequacies in digital material production^(21)^. In this sense, it is essential to critically screen the sources of authorship and the themes/issues addressed to ensure information reliability. But, in this study, it was interesting to note the quality of the material produced and the involvement of academics and professors in the authorship of materials that, at times, integrated monitoring activities, extension activities, scientific events or were linked to projects and graduate programs in different areas of knowledge. The participation of academics in the recording of educational videos contributes significantly to learning, as it encourages them to take the lead in the teaching-learning process^(22)^.

In this study, most of the videos meet the recommendations of experts who discuss teaching mediated by information technologies, as the recordings do not exceed 15–20 minutes^(23,24)^. Such criterion is recommended so that participants’ attention is not lost. Furthermore, YouTube videos of a few minutes can be more easily absorbed than a continuous 50-minute video^(19)^, unless other strategies are used in these to produce interaction with the audience.

Another observation regarding Iramuteq videos is that with the progression of the pandemic over time, the growing number of publications became evident. In this sense, some advantages can come from this process, such as: reaching an expressive audience; the possibility of pausing the videos while performing the software installation process step by step; the opportunity to access the content whenever the researcher, for instance, is using the program and has doubts about information. There is also the fact that they can check while watching the material and learning the content, having access not only to theoretical information, verifying the practical applicability of the software and, at the same time, developing technological skills for implementation.

The increase in the number of videos about Iramuteq, as well as the number of views (which have already surpassed the mark of 184,223 on the day of collection) allows us to infer that there is interest in this tool. Furthermore, it is believed that greater visibility and discussion of the topic on social media can result in an increase in scientific production using the software.

It is emphasized that Iramuteq performs lexicometry, i.e., the automatic treatment of texts from statistical calculations on the vocabulary of the analyzed corpus^(25)^. This methodological approach is especially used to identify trends, symmetries and discursive styles underlying patterns of association between words, expressions and concepts, reducing the material and making sense of the data cluster^(26)^.

Based on the terms of Lebart and Salem (1994), using statistics in textual data analysis can be decomposed, for discussion purposes, into four stages: research problem construction; production of data relevant to the problem and compatible with the methodological strategy; material statistical treatment; and interpretation of results^(5)^. In other words, the choice and decision to use the software must permeate the entire research planning and not only be restricted to the data processing stage.

It is noteworthy that there is still a lot to be explored about Iramuteq and that it may raise new videos, such as further explanations and details about DHC, since this analysis has been the most recurrent in research. About it, it is still possible to discuss the possibility of working sub-corpus of each class, how to graphically present the chi-square of each lexicon by class, how to identify the typical text segments and their scores (by lexicon and by class), among other aspects.

The research by specificity of groups can also be better detailed, suggesting, for example, its use to assess the difference between discourses from a gender perspective, since this type of analysis allows comparisons to be made based on their modalities^(6)^. It is essential to clarify that the choice for the use of one, two or more analysis techniques or the applicability of one or the other will depend on the characteristics of the problem and the research objectives^(26)^. After all, the simple allusion to software use in the materials produced, without a complete and adequate description of the types of analysis performed or the theoretical framework for the interpretation of the findings, weakens the validity of results^(2,5)^.

It is necessary to broaden discussions on matrix analyses, software use in bibliometric research, reflection on the need for researchers to anchor themselves in a theoretical framework to carry out data analysis, the differentiation of traditional content analysis, the lexical analysis developed by the software and the possibility of combining these types of analysis^(2,5)^, including as already suggested and developed in some researches. Moreover, there is a need to work tips on the presentation of research reports that used Iramuteq, to help the academic/researcher to transform the statistical results provided by the software into scientific writing.

It is believed that even the health condition related to the COVID-19 pandemic allows for a safe return to classrooms in the on-site modality. It is understood that teaching Iramuteq for use in qualitative research through YouTube videos. It is a relevant strategy, including the possibility of academics/researchers using this tool with technical and scientific capacity, sharing their experience in relation to handling the tool, the difficulties and/or facilities perceived, as well as the experiments developed. Even after overcoming this pandemic phase, such a strategy can be considered and articulated with moments of teaching, sometimes on-site, sometimes virtual.

As limitations, this study did not include videos broadcast internationally and considered data gathering on a single social network platform. However, this investigation can serve as an increment to other researches with a similar methodological design to the one developed in this investigation, but considering the gathering of French videos, since Iramuteq had its origins in France.

## CONCLUSION

The videos gathered explain how to install Iramuteq, the textual corpus preparation, the errors identified by users and methods to correct them, the lexical analysis developed by the program and how to use it in qualitative research. The content is described in a theoretical and practical way through the software interface presentation. The step-by-step demonstration of the software use demystifies the difficulties and clears doubts that might exist among those who intend and/or already use Iramuteq.

It is considered that, with the videos available, more researchers will have access to basic information for handling the software, thus being able to choose to use it in their research. Thus, there is an important contribution of social networks, with a focus on YouTube, as a communication and information technology strategy for the development of teaching in the emergency remote format and of research.

It was evident that few videos address Iramuteq use to process matrices. Furthermore, there are many features available in the program that are not even mentioned in the videos gathered. These gaps serve to suggest new videos with such approaches.

Furthermore, the on-screen article contributes by proposing that YouTube establish itself as a social media that by virtue of its easy and wide access. YouTube can be both a source of research and it can also raise dialogues between researchers, especially in a pandemic context. In other words, the platform use can be considered an important communication and information technology strategy for the development of teaching in the emergency remote format and for research.
